# Health outcomes of iron supplementation and/or food fortification in iron-replete children aged 4–24 months: protocol for a systematic review and meta-analysis

**DOI:** 10.1186/s13643-019-1185-3

**Published:** 2019-11-01

**Authors:** Dominic J. Hare, Sabine Braat, Bárbara R. Cardoso, Christopher Morgan, Ewa A. Szymlek-Gay, Beverley-Ann Biggs

**Affiliations:** 1 The Peter Doherty Institute for Infection and Immunity at The University of Melbourne, 792 Elizabeth Street, Melbourne, VIC 3000 Australia; 2 Department of Medicine at the Royal Melbourne Hospital and The University of Melbourne, 300 Grattan Street, Parkville, VIC 3052 Australia; 3 Melbourne Dementia Research Centre at The Florey Institute of Neuroscience and Mental Health and The University of Melbourne, 30 Royal Parade, Parkville, VIC 3052 Australia; 4 Centre for Epidemiology and Biostatistics, Melbourne School of Population and Global Health, University of Melbourne, Melbourne, VIC 3010 Australia; 5 Institute for Physical Activity and Nutrition, School of Exercise and Nutrition Sciences, Deakin University, Locked Bag 20000, Geelong, VIC 3220 Australia; 6 Burnet Institute, 85 Commercial Road, Melbourne, VIC 3004 Australia; 7 Melbourne School of Population and Global Health, The University of Melbourne, 235 Bouverie Street, Carlton, VIC 3053 Australia; 8 School of Public Health and Preventive Medicine, Monash University, 553 St Kilda Road, Melbourne, VIC 3004 Australia; 9 Victorian Infectious Diseases Service, Royal Melbourne Hospital, 300 Grattan Street, Parkville, VIC 3052 Australia

**Keywords:** Critical windows, Dietary iron, Infant nutrition, Iron deficiency, Iron deficiency anaemia, Iron fortification, Neurodevelopment

## Abstract

**Background:**

Direct supplementation or food fortification with iron are two public health initiatives intended to reduce the prevalence of iron deficiency (ID) and iron deficiency anaemia (IDA) in 4–24-month-old infants. In most high-income countries where IDA prevalence is < 15%, the recommended daily intake levels of iron from supplements and/or consumption of fortified food products are at odds with World Health Organisation (WHO) guidelines that recommend shorter-term (3 months/year) supplementation only in populations with IDA prevalence > 40%. Emerging concerns about delayed neurological effects of early-life iron overexposure have raised questions as to whether recommended guidelines in high-income countries are unnecessarily excessive. This systematic review will gather evidence from supplementation/fortification trials, comparing health outcomes in studies where iron-replete children did or did not receive additional dietary iron; and determine if replete children at study outset were not receiving additional iron show changes in haematological indices of ID/IDA over the trial duration.

**Methods:**

We will perform a systematic review of the literature, including all studies of iron supplementation and/or fortification, including study arms with confirmed iron-replete infants at the commencement of the trial. This includes both dietary iron intervention or placebo/average dietary intakes. One reviewer will conduct searches in electronic databases of published and ongoing trials (Medline, Web of Science, Scopus, CENTRAL, EBSCO [e.g. CINAHL Complete, Food Science and Technology Abstracts], Embase, ClinicalTrials.gov, ClinicalTrialsRegister.eu and who.it/trialsearch), digital theses and dissertations (WorldCat, Networked Digital Library of Theses and Dissertations, DART-Europe E-theses Portal, Australasian Digital Theses Program, Theses Canada Portal and ProQuest). For eligible studies, one reviewer will use a data extraction form, and a second reviewing entered data for accuracy. Both reviewers will independently perform quality assessments before qualitative and, if appropriate, quantitative synthesis as a meta-analysis. We will resolve any discrepancies through discussion or consult a third author to resolve discrepancies. The Preferred Reporting Items for Systematic Reviews and Meta-Analyses statement will be used as the basis for reporting.

**Discussion:**

Recommended iron supplementation and food fortification practices in high-income countries have been criticised for being both excessive and based on outdated or underpowered studies. This systematic review will build a case for revisiting iron intake guidelines for infants through the design of new trials where health effects of additional iron intake in iron-replete infants are the primary outcome.

**Systematic review registration:**

PROSPERO CRD42018093744.

## Background

### Rationale

Adequate dietary iron intake during early life is crucial for several developmental processes, particularly in the brain. Exclusively breastfed term infants have access to sufficient levels of iron for the first 4–6 months of life, during which 20–40%, (~ 50–100 mg) of total body iron at birth is recycled from excess haemoglobin (Hb) and stored for later use [1], with an additional ~ 0.15 mg absorbed from breastmilk containing around 0.5 mg/L of iron per day [2]. Foetal iron demand is highest during the third trimester [3]; thus pre-term and low birthweight infants are generally considered at higher risk of neurological deficits arising from iron deficiency and routinely receive supplemental iron during the neonatal period [4].

The final 18 months of the first 1000 days (conception to 2 years of age) represent a critical window of nutrition-dependent development, primarily in the central nervous system [5]. Iron supports myelination, establishment and consolidation of neurotransmitter pathways and the substantial metabolic needs rapidly proliferating neural networks [6]. Insufficient supply of iron during this period has marked and well-characterised effects on neurodevelopment [7], including weaker cognitive, motor and social development compared to iron-replete infants [8]. By comparison, relatively little is known about the effects of chronic dietary iron overexposure during this period, though emerging concerns regarding delayed neurotoxic effects of iron-mediated oxidative stress have sparked new debate among biochemists, nutritionists and paediatricians about appropriate intake during this critical window [9–11].

Historically, most high-income countries implemented both targeted food fortification programs, such as the addition of inorganic iron to infant formula [12], and broader open-market fortification of staple cereal products [13] to address the high prevalence of iron deficiency (ID) and iron deficiency anaemia (IDA) in both infants and the wider populations during the mid-twentieth century. By 2011, the number of children 6–59 months meeting the diagnostic criteria for IDA (Hb < 110 g/L [14]) in participating high-income countries had dropped to < 15% [15]. While aggressive infant formula fortification regulations have likely contributed to this decrease—the American Academy of Paediatrics (AAP) Committee on Nutrition (CoN) has supported the use of formula containing 10–12 mg/L of iron for over three decades [16]—so has increased availability of complementary foods, including iron-fortified infant cereals and staple foods [10]. The vast majority of infants and toddlers in the USA already receive adequate nutrient intake through diet alone [17]. In high-income European countries, guidelines for formula fortification are more conservative: non-breastfed term infants are advised to consume preparations containing 4–8 mg/L to 6 months with no set levels for 6–24-month-old children, based on a lack of evidence supporting optimal iron concentrations and acknowledgement of concerns regarding long-term adverse outcomes arising from excessive brain iron levels [18]. Both North American and European guidelines are substantially more hawkish than those issued by the World Health Organisation (WHO) in 2016 that encompass all countries, regardless of gross domestic product. The WHO recommends daily oral supplementation for children aged 6–23 months with 10–12.5 mg of elemental iron for no longer than three consecutive months a year, and only in areas where IDA prevalence is > 40% and malaria is not endemic [19].

By contrast, the AAP CoN also recommends augmentation with direct supplementation to 12 months, followed by the introduction of multivitamin preparations to 36 months ‘if iron needs are not being met’ [16]. Herein lies a major outstanding question: while daily iron intake from complementary foods is substantially more variable than regular formula consumption, does the consumption of iron-rich foods, particularly those containing highly bioavailable haem, compound average intake to a point where the need for fortified formula and/or oral supplementation becomes unnecessary? Predictive modelling of a scenario in the USA where legislation requiring open-market fortification of staple foods is abolished suggests a modest increase in IDA prevalence in young children, though still well below 10% [20]. Screening for IDA and IDA risk (as altered haematological markers indicative of ID without anaemia) is recommended by the APP every 12 months to identify infants in need of adjuvant supplementation [16]. We argue that this approach has limited clinical utility, as there is no global consensus regarding cut-off levels of non-Hb markers used to identify ID [21]. Twelve-month intervals between screening provide limited information on time trends that would indicate iron stores are being depleted, and ID itself is asymptomatic and may not be pathological if markers remain stable over a set period. Concerning the latter point, an interesting theory proposed by Quinn [22] and supported by recent trials in Kenya [23, 24] where bacterial gastroenteritis is endemic posits that ID during the 6–24-month critical window is an evolutionary mechanism intended to limit the proliferation of pathogenic iron-dependent gut bacteria [22].

There is no question that adequate dietary iron intake is critical for normal neurodevelopment and overall healthy growth, though reassessment of long-standing policies promoting potentially excessive intake in a high-income setting are well overdue. A recent systematic review and meta-analysis of 35 trials involving over 40,000 infants from 4 to 23 months lacked sufficient statistical power to identify any neurodevelopmental benefit of daily iron supplementation [25]; and the most extended prospective cohort (*n* = 437; 43% attrition) assessed to date reported that children fed ‘low’ (2.3 mg/L) iron formula from 6 to 12 months outperformed those receiving AAP CoN-comparable 12.7 mg/L fortified preparations in all measures of neurodevelopment at 10 years, including spatial memory, visual-motor integration and intellectual ability [26]. Although far from conclusive, this does raise some concerns about potential delayed adverse long-term health outcomes beyond those characterised in IDA. This systematic review and meta-analysis will examine effects of supplementary iron intake via direct supplementation or food fortification in non-IDA infants on haematological indices of iron stores, growth, neurodevelopment and adverse health effects. This study will help to determine if more research focussing on iron-replete infants in contemporary high-income settings are needed to inform revised nutritional guidelines. The ultimate goal is to establish ideal intake levels that sustain infant IDA levels at their current low rate and minimise any potential long-term risks arising from overexposure to iron during this critical window of neurodevelopment.

### Research questions and objective

#### Research questions

1. What effect does iron supplementation and/or fortification of infant food products have on health outcomes in iron-replete children aged 4–24 months, compared to iron-replete children not receiving supplemental dietary iron?
2. Do iron-replete children in upper-middle and high-income countries show evidence of iron deficiency (by established haematological measures) when not receiving supplemental dietary iron?

#### Objective

The objective of this systematic review and meta-analysis is to review the literature and identify all potentially relevant clinical trials of iron where at least one study arm included infants aged within the 4–24-month window who were not classified as ID or IDA (iron-replete) at trial initiation. These data will be assessed for quality and used to examine and compare the effects of augmented iron intake on developmental outcomes, adverse events and haematological indices of iron status with non-supplemented infants.

## Methods

This protocol is detailed according to the recommendations of the National Institute for Health and Clinical Excellence (NICE; United Kingdom) [27] and the Cochrane Handbook [28], and it is reported according to the Preferred Reporting Items for Systematic Reviews and Meta-Analyses Protocols (PRISMA-P) statement (Supplementary Material).

### Context

We will include trials conducted in any country or jurisdiction, rural and urban sites and in both community and health care settings. We will include countries that differ in their national policy for iron fortification, including countries with no active policy.

### Eligibility criteria

#### Types of studies

Crossover, individually and cluster-randomised or quasi-randomised (provided the allocation methods are clearly described) controlled trials will be included, as well as follow-up studies of previous trials. We will only include trials for which the intervention was applied directly towards the child; studies attempting to improve child health through antenatal or postnatal intervention for the mother will not be included. Crossover studies will only be included in the first half of the trial and analysed separately. For the primary question only (research question 1), included randomised and quasi-randomised controlled studies must involve a total duration of 4 weeks or longer of supplementation or fortification. Also, non-randomised trials—specifically individual and cluster non-randomised controlled trials—individual and cluster-controlled before-and-after trials, prospective cohort studies and single-arm studies will be included for the secondary question (research question 2). Cross-sectional studies, case reports, animal studies, editorials, letters, narrative reviews and surveys will not be included. We will include both published and unpublished studies.

#### Types of participants

Infants born at term (currently defined by the American College of Obstetricians and Gynaecologists as 37 weeks to 41 weeks and 6 days gestation [29]) aged 4–24 months who are classified as non-anaemic at study commencement, per the definition of anaemia used in each trial. While acknowledging that pre-term infants are more susceptible to acute effects of iron overload [1] and current practice should also be assessed soon in terms of effective dose, near-universal supplementation of premature and low birth weight infants excludes these participants.

We will include participants fulfilling the inclusion and exclusion criteria from all countries, settings (e.g. rural or urban), socioeconomic status and background malaria endemicity (iron deficiency is believed to protect against proliferation of the *Plasmodium falciparum* parasite, while conversely, malaria is a substantial contributor to global anaemia burden, causing a non-iron-related haemolytic anaemia [30]). For the secondary question, we will examine a subset of the full dataset, including only participants in upper-middle and high-income countries with reported Hb concentrations.

#### Types of interventions

Supplemental dietary iron is defined as any source of iron taken orally as part of a randomised controlled trial where the amount of iron exposure is either explicitly stated or can be established from the presented information. Interventions to be included are direct oral supplementation (drops, syrups, etc.) or fortified infant formula or food products (milled grains, cereal products, etc.) provided mean daily iron intake can be calculated based on reported volume or caloric consumption data. Study arms where mean daily iron intake cannot be obtained or calculated will be excluded.

All non-haem iron additives will be considered, including ferrous sulphate, ferrous fumarate, ferrous gluconate, carbonyl iron and colloidal iron will be included. Protein-bound iron will also be included—human breast milk contains little inorganic iron [31], with the majority bound to lactoferrin [32]. The frequency of supplementation or fortification will be considered for total iron intake but will not preclude any study from inclusion. Iron may have been delivered as a liquid, tablet, capsule, lozenge or dispersible tablet.

Trials of multiple vitamins and micronutrients will be excluded, except those that examine the additional effect of iron with consistent levels of other vitamins and minerals maintained across all treatment groups. Any co-intervention believed to influence iron uptake (e.g. ascorbic acid, zinc, etc.) will also be included in all groups so as the sole intervention variable between arms is total iron intake.

### Information sources

The following online databases will be searched from inception to 30 June 2019:

- Medline via Ovid including Epub Ahead of Print, In-Process and other non-indexed citations
- Web of Science (including Conference Proceedings Citation Index-Science and Biosis Previews)
- Scopus
- Cochrane Central Register of Controlled Trials (CENTRAL)
- EBSCO (Academic Search and Applied Science and Technology databases)
- Embase via Ovid
- ClinicalTrials.gov
- clinicaltrialsregister.eu
- who.int/trialsearch

Forward citation tracing will be performed during the initial database search where available, including Medline, Web of Science and Scopus.

Digital theses will be searched for on:

- WorldCat
- Networked Digital Library of Theses and Dissertations
- DART-Europe E-theses Portal
- Australasian Digital Theses Program
- Theses Canada Portal
- ProQuest-Dissertations and Theses

Only studies in English will be included, and no other limitations will be applied to the search.

### Search strategy

We will search with combinations of the following terms:

- Iron (ferrous* ferric* trace element* micro*nutrient*)
- Supplementation (supplement* drops* liquid*)
- Fortification (fortif* formula*)
- Infants (infant* child* baby babies newborn* toddler* pre-school* preschool*)
- Anaemia (anemia anemic anaemic)

The present draft of the search strategy to be used for Scopus is:

- ((TITLE-ABS-KEY(iron OR ferric* OR ferrous* OR trace element* OR micro*nutrient*)) AND (TITLE-ABS-KEY(infant* OR child* OR baby OR babies OR newborn* OR neonat* OR toddler* OR preschool* OR pre-school*)) AND (TITLE-ABS-KEY(supplement* OR drops OR liquid OR fortif* OR formula* OR diet* therap*)) AND (TITLE-ABS-KEY(anemi* OR anaemi*)) AND (TITLE-ABS-KEY(rct OR placebo* OR trial* OR randomi* OR cross-over OR cross over OR before-after OR before and after OR cohort)))

### Study records

#### Data management

Full texts will be collated in the *Papers* desktop application (ReadCube, USA) before entering data into *Review Manager* (*RevMan*) v5.3 [33]. Data will be extracted or obtained from studies using a data extraction form. The form will be piloted on a small number of study reports (approx. 10 eligible studies published between 2000 and 2013; based on included studies reported in Pasricha et al. [25]) and modified if necessary.

#### Selection and data collection process

The selection of studies will be made by merging search results from each database, removing any duplicates, title assessment and examination of full-text reports according to inclusion criteria. For studies judged to be eligible or potentially eligible for inclusion, the full-text articles will be sourced and assessed by two reviewers. Review authors will extract data from eligible reports using the form. One author will enter data into *RevMan*, and the second author will carry out checks for accuracy. We will resolve any discrepancies through discussion or consult a third author. Any discrepancies in the classification that arise during this process will be resolved by consensus between the two reviewers and a third reviewer. For studies that progress to the full-text screening stage, we will record the reason that studies were excluded.

If there are cases where it is unclear whether the inclusion criterion is met, we will attempt to contact the study corresponding author for clarification; if no response is received within 4 weeks of the request, or the requested information is not provided, the information within the full-text article will be used to decide on the eligibility of the study.

A diagram will be created to report the flow of studies through the systematic review.

### Data items

For each study, we will collect data on source, study authors and contact information, design, setting, participants, the interventions applied, reported comorbidities outcomes measured, a full description of the results including the stated outcomes listed above, national policies on iron fortification in place at the time of the study, funding sources and other relevant outcomes identified using the standardised collection form. We will attempt to find relevant missing information by contacting the primary/corresponding author of the study. Extracted data will be summarised in tables. If any data extraction discrepancies arise, these will be resolved by discussion and consensus among the review team.

### Outcomes and prioritisation

#### Primary outcomes

We will examine the effect of supplemental dietary iron on health outcomes associated with IDA in infancy. These include:

1. Measures of physical growth, including weight (kg), weight-for-age (*Z*-score), length (cm), length-for-age (*Z*-score), weight-for-length (*Z*-score), stunting and wasting.
2. Neurodevelopmental indices, where available, including (but not limited to) scores on validated developmental tests for example Bayley scales, pre-school language scale, child behaviour checklist, Denver developmental screening test, Fagan test of infant intelligence, Infant behaviour questionnaire, toddler behaviour questionnaire, cognitive tasks and motor testing, infant attachment/bonding and physical activity/attentiveness (as measured by trialists).
3. Anaemia (defined as the Hb concentration cut-off used in the study, adjusted by altitude and age as appropriate) and haematological iron indices, including blood Hb concentration (g/L), ferritin (FTN) concentration (ng/mL), transferrin (Tf) concentration (mg/mL) and saturation (Tf_sat_; %), soluble transferrin receptor (TfR; mg/mL) and the zinc protoporphyrin (ZPP) to haem ratio. Note that study-specific Hb concentrations for IDA diagnosis are used in place of the < 110 g/L level set by the WHO and most commonly used in clinical practice [14] to include trials where lower Hb cut-offs were used. This is justified on the basis of preliminary reports of asymptomatic infants with sub-110 g/L Hb levels that clinically identify them as IDA [34] and calls for a re-evaluation of current diagnostic criteria for infants [18].
4. Safety and adverse health effects (including vomiting, fever, diarrhoea [both prevalence and incidence], infectious disease, constipation, hospitalisation or non-regular clinic visits, morbidity from iron-related poisoning and mortality).
5. Other micronutrient intake and status where an effect from iron is suspected (calcium, zinc, vitamin A, etc.).

#### Secondary outcomes

To examine the effects of iron supplementation and/or food fortification on clinical markers of IDA, we will report:

6. Changes from baseline in haematological iron indices, including blood Hb concentration (g/L), FTN concentration (ng/mL), Tf concentration (mg/mL) and Tf_sat_ (%), and soluble TfR.

Data will be extracted from trials with a study arm meeting the inclusion criteria that were performed in upper-middle and high-income countries. Classification of economies is based on that used by the World Bank for the 2018/19 fiscal year [35].

Timing of outcome assessment will be based on the duration of the intervention and trial follow-up after completion of the intervention period. We will perform a subgroup analysis where studies are segregated by the duration of the intervention and outcomes defined as the difference between trial completion and commencement, compared against trial length (rate of change). We will also examine measured outcomes following trial completion (e.g. those measured up to 6 months following completion of the trial, those measured 6–12 months following completion of the trial and those measured more than a year following completion of the trial).

### Risk of bias in individual studies

Statistical analyses will be undertaken using *RevMan* and *Stata Statistical Software* v15.1 (StataCorp, USA) [36]. Risk of bias will be assessed by two review authors independently. We will resolve any discrepancies through discussion or consult a third author to resolve discrepancies.

For the included randomised controlled trials, risk of bias will be assessed using the *RoB 2.0* tool [37], specific for the type of trial (individually randomised parallel group trial, cluster-randomised parallel group trial, individually randomised cross-over trial) and each bias domain will be assessed for each outcome within each included study. The following domains will be classified:

1. Bias arising from the randomisation process
2. Bias due to deviations from intended interventions
3. Bias due to missing outcome data
4. Bias in measurement of the outcome
5. Bias in the selection of the reported result

For cluster-randomised parallel group trials only, we will assess an additional domain:

6. Bias arising from the timing of identification and recruitment of individual participants with the timing of randomisation

Risk of bias in each domain will be classified as low, high or some concerns. We will reach an overall risk of bias judgement as low, high or some concern for each outcome within each included study across all domains according to the process outlined for *RoB 2.0* [37].

For the included quasi-randomised and non-randomised studies, risk of bias will be assessed using the Risk Of Bias In Non-randomised Studies-of Interventions tool (ROBINS-I) [38] and each bias domain will be assessed for each outcome within each included study. The following domains will be classified:

1. Bias due to confounding
2. Bias due to the selection of participants into the study
3. Bias in the classification of interventions
4. Bias due to deviations from intended interventions
5. Bias due to missing data
6. Bias in measurement of outcomes
7. Bias in the selection of the reported result

Risk of bias in each domain will be classified as low, moderate, severe, critical or no information. We will reach an overall risk of bias judgement as low, moderate, severe, critical or no information for each outcome within each included study across all domains according to the process outlined for ROBINS-I [38].

For the included single-arm studies, bias will be assessed using a modified ROBINS-I approach [38] described by Jullien et al. [39]; and the Quality in Prognosis Studies tool [40] and checklist used by NICE [27] with the following domains:

1. The study sample represents the population of interest concerning key characteristics, sufficient to limit potential bias to the results.
2. Loss to follow-up is unrelated to key characteristics (that is, the study data adequately represent the sample), sufficient to limit potential bias.
3. The prognostic factor of interest is adequately measured in study participants, sufficient to limit potential bias.
4. The outcome of interest is adequately measured in study participants, sufficient to limit bias.
5. Important potential confounders are appropriately accounted for, limiting potential bias for the prognostic factor of interest.
6. The statistical analysis is appropriate for the design of the study, limiting the potential for the presentation of invalid results.

If a standardised tool for single-arm studies was to be released during the conduct of our systematic review, we will adopt this tool and conduct the risk of bias assessment for single-arm studies accordingly.

### Data synthesis and analysis

#### Criteria for quantitative synthesis of data

If the outcome is reported for more than one trial, we plan to conduct meta-analysis but will report only if no substantial statistical heterogeneity is detected. We will quantify heterogeneity using the *I*^2^ test; substantial heterogeneity is considered when *I*^2^ exceeds 50%.

#### Summary measures, data handling and methods for combining data

For continuous outcomes, we will use mean differences (MD) with 95% confidence intervals (CIs) if outcomes were measured in the same way or standardised mean differences (SMD) and 95% CIs if different scales were applied across studies to measure the same outcome. For binary outcomes, we will use the risk ratio (RR) (primary outcomes) or proportion (secondary outcomes) and 95% CIs.

#### Unit of analysis

We will use the participant as the unit of analysis. For cluster or cross-over randomised controlled trials, we will take the level of randomisation into account for cluster trials and use only data of the first treatment period for cross-over trials. In the case of multi-arm trials, we will combine relevant groups to create two arms to allow for single-pair comparisons. For continuous outcomes, the feasibility hereof depends on the availability of the sample size, mean and standard deviation within each arm.

#### Missing data

We will contact the primary/corresponding authors of the studies in case of missing (summary) data that we cannot derive from the available data or in case of insufficient methodological detail. We will record the attrition (e.g. lost to follow-up, drop-out, withdrawal of consent) for included studies and review the methodology used by the authors to assess the adequacy in dealing with missing data. We will perform an available case analysis (includes only those participants whose outcome data are known) and imputed case analysis (with missing values imputed using specific assumptions).

#### Assessment of heterogeneity

We anticipate that there may be heterogeneity between studies attributable to methodological (i.e. formulations, implementation/ delivery strategies), study type (randomised/quasi-randomised), population (baseline Hb and iron status, setting) and implementation differences, as well as statistical heterogeneity. Clinical heterogeneity will be explored via the characteristics of the included studies. Statistical heterogeneity will be examined visually using forest plots and statistically using the Cochran’s Q test, the *I*^2^ statistic and tau^2^.

#### Subgroup analyses

We plan several subgroup analyses for the primary question:

1. By gross national income per capita (by World Bank criteria) of the country in which trial was performed: high-income, upper-middle income, or other.
2. By national food fortification policy: mandatory iron fortification, voluntary iron fortification, no iron fortification.
3. By baseline iron stores (by serum ferritin concentration according to WHO guidelines for children < 5 years): sufficient (≥ 12 μg/L), insufficient (< 12 g/L) or not reported.
4. By baseline Hb (by WHO guidelines): non-anaemic (≥ 110 g/L), mild anaemia (100–109 g/L), moderate anaemia (70–99 g/L), severe anaemia (< 70 g/L) or not reported.
5. By route of administration: direct supplement, fortified food.
6. By mean dose of iron supplementation per day: 12.5 mg or less, > 12.5 mg–< 3  mg, 31–59 mg, 60 mg or higher.
7. By duration of iron supplementation: 1–3 months, greater than 3 months.
8. By duration of follow-up: < 6 months, 6–12 months, greater than 12 months.
9. By breastfeeding status: breastfeeding, weaned, not reported.
10. By sex of the participants: male, female, not reported
11. By regional malaria endemicity [41], where *Pf*PR = *Plasmodium falciparum* parasite rate, and *Pf*API = *P*. *falciparum* annual parasite incidence (intense stable endemic [*Pf*PR ≥ 40%], moderate stable endemic [*Pf*PR 5.1–33.99%], unstable endemic [*Pf*PR ≤ 5%, *Pf*API = 0.01–0.1%], non-endemic [*Pf*API < 0.01%]).

We will explore the Forest plots visually and use statistical tests to investigate subgroup differences formally.

#### Sensitivity analysis

We plan a number of sensitivity analyses to investigate the robustness of results to decisions and assumptions on:

1. Studies without premature or low birth weight participants
2. Studies with inactive comparator arm only
3. Studies with low risk of bias only
4. Studies that were randomised only
5. Studies that were single-arm only (secondary objective only)
6. Assumptions made on cluster-randomised controlled trials (e.g. intra-cluster correlation)
7. Assumptions made on missing data imputation

#### Quantitative synthesis

We will analyse the data with both fixed-effect and random-effects models and will present the results of the random-effects meta-analysis. If the results of both models differ, only then will we also report the fixed-effect meta-analysis.

For the primary question, the meta-analysis will provide an overall estimate of the effect of iron supplementation or fortification compared to no intervention/placebo for outcomes based on comparative data only. For the secondary question, it will provide an estimate of the outcomes based on data from comparative (separate arms) and non-comparative (single-arm) trials.

#### Meta-bias(es)

We anticipate that studies in infants may have small sample sizes and be less likely to produce significant findings, which might have resulted in publication bias or time-lag bias. Possible reporting bias of included studies will be assessed using funnel plots if ten or more studies are included in a particular analysis. Statistical testing will be performed for funnel plot asymmetry.

#### Confidence in cumulative evidence

If appropriate, we will present a ‘Summary of Findings’ table for the quantitative outcomes. In this case, the quality and strength of the body of evidence will be assessed based on the Grading of Recommendations Assessment, Development and Evaluation (GRADE) method [42].

## Discussion

It has widely been assumed that moderately excessive dietary iron intake during development has no acute or chronic adverse health effects, aside from conflicting reports of mild gastrointestinal symptoms. However, the long-term health effects, particularly regarding neurodevelopment, remain unclear. The WHO has identified that the collection of ‘additional data on the safety of iron supplementation in non-anaemic or non-iron-deficient children’ should be a research priority, and that ‘additional long-term studies on functional outcomes (e.g. cognitive and motor development)’ should be conducted [19]. For a child to be considered iron-replete or non-ID/IDA, the most widely used clinical definition is blood Hb levels > 110 g/L. However, other haematological indices, including transferrin saturation and ferritin levels, can also provide a complete picture of iron status.

The strategy to meet our primary objective has been designed to compare the effect of iron supplementation or fortification of infant food products to no intervention/placebo on health outcomes in iron-replete children aged 4–24 months. To address the WHO’s identified research priorities, the effects of iron supplementation and/or fortification on (i) weight and growth, (ii) neurodevelopmental outcomes and (iii) haematological indices of iron status in children who are considered iron-replete (Hb > 110 g/L) will be collected via this systematic review and, if possible, undergo meta-analysis.

We have also designed our secondary objective to determine if iron-replete children in upper-middle and high-income countries who receive no supplemental dietary iron show evidence of a change in Hb concentration over time by established measures. With growing concern about delayed effects of unnecessary iron intake during critical windows of development, we believe this study is timely and will provide an evidence-based case for revisiting nutritional guidelines in these countries, some of which are based on public health policies that were initially devised over half a century ago.

## Data Availability

Not applicable at this stage. Once the systematic review and meta-analysis have been completed, data will be made available as supplementary files.

## Abbreviations

AAP: American Academy of Paediatrics
AAP-CoN: American Academy of Paediatrics Committee on Nutrition
CENTRAL: Cochrane Central Register of Controlled Trials
CI: Confidence intervals
FTN: Ferritin
GRADE: Grading of Recommendations Assessment, Development and Evaluation
Hb: Haemoglobin
ID: Iron deficiency
IDA: Iron deficiency anaemia
MD: Mean difference
NICE: National Institute for Health and Clinical Excellence
P*f*API: *P*. *falciparum* annual parasite incidence
P*f*PR: *P*. *falciparum* parasite rate
PRISMA-P: Preferred Reporting Items for Systematic Reviews and Meta-Analyses Protocols
RoB: Risk of bias tool
ROBINS-I: Risk Of Bias In Non-randomised Studies-of Interventions tool
RR: Risk ratio
SMD: Standard mean differences
Tf: Transferrin
TfR: Transferrin receptor
Tf_sat_: Transferrin saturation
WHO: World Health Organisation
ZPP: Zinc protoporphyrin
